# Intrathoracic Gastric Volvulus Following Laparoscopic Fundoplication: A Case Report and Review of the Literature

**DOI:** 10.7759/cureus.77452

**Published:** 2025-01-14

**Authors:** Mahdi Albandar, Jumana A Fatani

**Affiliations:** 1 Department of Surgery, King Saud Medical City, Riyadh, SAU; 2 Department of Surgery, Specialized Medical Center, Riyadh, SAU

**Keywords:** fundoplication, gastric bypass, hiatus hernia, strangulated, volvulus

## Abstract

Gastric volvulus is a fatal complication that rarely occurs after Nissen fundoplication with organoaxial volvulus being the most common type. We present a case of a 36-year-old male with a surgical history of Nissen fundoplication with hiatal repair three years prior to presentation. He presented with shortness of breath, abdominal pain, and obstipation. CT scan showed no passage of the oral contrast towards the stomach. The stomach is seen completely herniated into the chest suggesting rotation along its long axis worrisome for organoaxial volvulus. Intraoperative findings were a strangulated stomach through the diaphragmatic defect and migration of the entire stomach into the chest, at which division of the diaphragm and retrieval of the stomach after extensive adhesiolysis were successfully performed. The stomach was reduced back to the abdomen but was completely black and gangrenous; thus, total gastrectomy and Roux-en-Y gastric bypass were done, and the hiatus hernia was repaired with chest tube placement. Acute gastric volvulus after laparoscopic fundoplication is a surgical emergency that presents diagnostic challenges. It should be considered in the differential diagnosis of patients experiencing recurrent vomiting following fundoplication.

## Introduction

Gastric volvulus is a fatal complication that rarely occurs after Nissen fundoplication [1]. It can present with mild symptoms of abdominal pain and vomiting to severe complications such as stomach strangulation and death [1]. The standard presentation of acute gastric volvulus is the triad of retching, epigastric pain, and difficulty or inability to insert a nasogastric tube (NGT) [1,2]. The difficulty in diagnosing volvulus prior to surgery is often due to the condition's rarity and atypical radiological features [3]. Surgical treatment includes reducing the volvulus and taking measures to prevent recurrence [2]. We present a case of acute intrathoracic gastric volvulus after laparoscopic fundoplication managed by total gastrectomy and Roux-en-Y gastric bypass.

## Case presentation

A 36-year-old male with a history of Nissen fundoplication and hiatal repair for gastroesophageal reflux disease (GERD) was performed three years prior to presentation. The patient presented with epigastric and left upper abdominal pain and shortness of breath for five days, associated with nausea, anorexia, and vomiting, with complete obstipation for three days. He sought medical advice at another hospital, where an X-ray was done and showed a picture of gut obstruction, and recommended to be evaluated at his primary hospital. He came to our hospital at the gastrointestinal clinic and was advised to go to the emergency room (ER). Upon assessment in the ER, the patient was sick-looking, orthopneic, on 5 Liter (L) O2, tachycardiac, and afebrile, and blood pressure was maintained. No air entry on left lower lung. The abdomen was distended with tenderness at the epigastrium. Bowel sound was very sluggish. Per rectal (PR) showed an empty rectum. An NGT was inserted and 2L of massive dark red blood with “coffee ground” content was aspirated. Laboratory results revealed leukocytosis.

**Figure 1:**
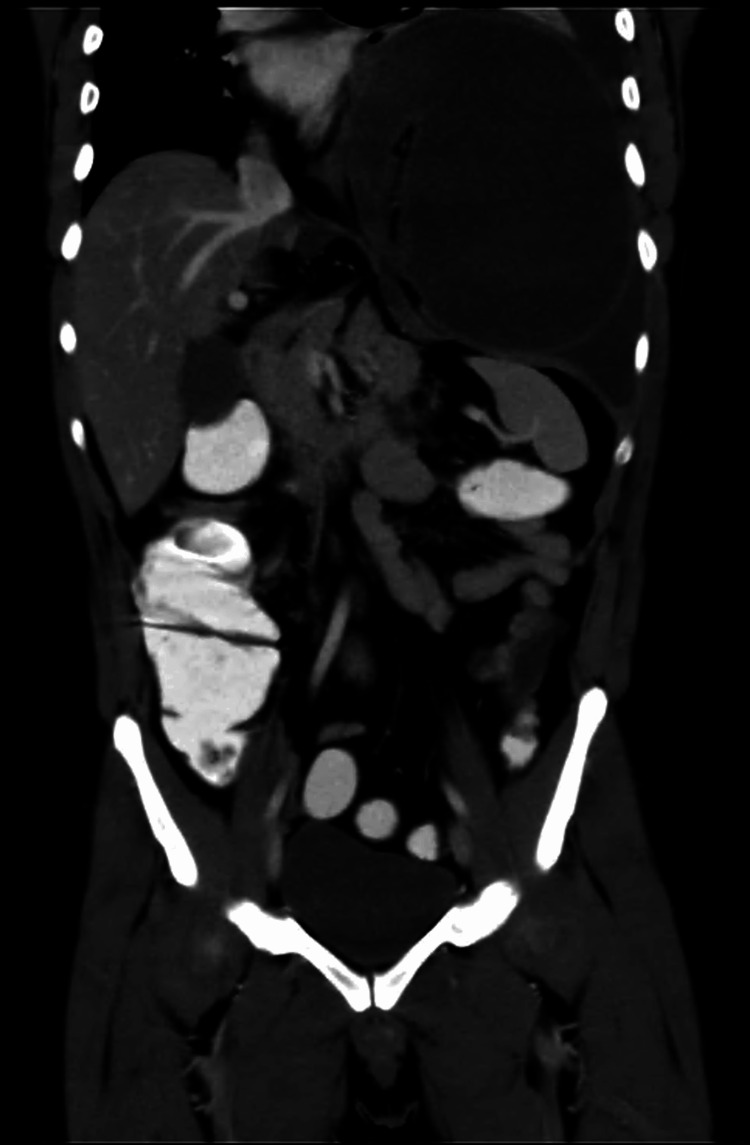
Coronal view of CT scan showing the stomach herniated into the chest.

CT of the abdomen showed the esophagus is distended and filled with oral contrast with no passage of the oral contrast toward the stomach. The stomach is seen completely herniated into the chest; it’s fluid-filled and dilated, suggesting rotation along its long axis, which was worrisome for organoaxial volvulus (Figure 1). Subsequent imaging revealed a moderate to large left-sided pleural effusion, consolidative segmental and subsegmental areas of consolidation, and collapse of the left lung. There was a right-sided mediastinal shift, along with a small right pleural effusion. Additionally, a trace of free fluid with fat stranding was noted in the left upper abdominal quadrant. The patient was shifted to ICU, and gastroenterology was contacted. An upper endoscopy was done, and an element of gastric mucosal ischemia was observed, with blood oozing and gastric volvulus.

The patient was shifted emergently to the operating room for diagnostic laparoscopy. Intraoperatively, a narrow diaphragmatic defect was seen with the migration of the entire stomach inside the pleura cavity, causing adhesion at the pleura space with a narrow defect at the diaphragm. Fundoplication at the right side was still attached to the crus; however, the entire greater curvature migrated upward. Also, there were whitish deposits all over the mesentery, which was worrisome for tuberculosis (TB) and biopsies were taken. Widening of the defect and hardly the herniated stomach was retrieved back to the abdominal cavity, which was completely black and ischemic from the cardia and extending down to the pylorus. The decision was made to do a total gastrectomy and Roux-en-Y gastric bypass and the diaphragmatic defect was repaired. A chest tube was inserted at the left pleura space and fixed to an underwater seal. Oxygen saturation improved to 100% after inflation with full lung expansion. Postoperatively, the patient was shifted to ICU and was extubated the following day. Gastrografin was done the following day and showed free contrast passage with no contrast leak or delay. The patient was observed over three days and then started on a clear fluid diet, which was tolerated well by the patient. Eight days postop, he was discharged with no active complaints. The patient was followed in the clinic with complete recovery and no complaints. The final pathology report showed acute necrotizing gastritis/gastric gangrene secondary to strangulation by the diaphragmatic hernia. The omentum biopsy was consistent with acute necrotizing inflammation. The pleural adhesion biopsy revealed partially infarcted fatty tissue.

## Discussion

Gastric volvulus presents with three types based on the axis of rotation, namely organoaxial, mesenteroaxial, and a combination of both [1,2]. Organoaxial volvulus is the most common, occurring at a rate of 59%, involves the rotation of the stomach along its long axis, and is often associated with hiatus hernia [1,2]. Strangulation has been documented in 5-28% of gastric volvulus cases [1]. The causes of gastric volvulus can be categorized into primary and secondary etiologies. Primary gastric volvulus typically results from the laxity of the supportive ligaments of the stomach, allowing the stomach to twist along its mesentery. Secondary volvulus can be influenced by conditions such as hiatal hernia, traumatic diaphragmatic rupture, eventration of the diaphragm, abdominal adhesions, damage to the ligaments anchoring the stomach in the abdomen, intrinsic gastric pathology, or masses outside the stomach [1]. Foreign bodies, such as stitches, can lead to adhesion and subsequent gastric volvulus [3,4]. Postoperative temporary traumatic edema may predispose the development of gastric volvulus [3].

Acute gastric volvulus following laparoscopic fundoplication represents a surgical emergency and poses challenges in diagnosis [2]. Early diagnosis is paramount to prevent serious and potentially fatal complications [2]. A combination of a high index of clinical suspicion and radiologic investigations leads to the diagnosis [2]. Failure to diagnose volvulus prior to surgery can be attributed to this condition's rarity and atypical radiological features [3]. Elevated serum amylase levels are commonly observed, and a CT scan is crucial for diagnosing gastric volvulus [1]. In some cases, gastric volvulus may spontaneously reduce, leading to potential intermittent torsion and a missed diagnosis [2]. Initial attempts at decompression through nasogastric and gastroscopic procedures should be made [2]. Surgery aims to reduce the volvulus and prevent recurrence, involving techniques such as volvulus reduction, hernial sac reduction, hiatal repair, fundoplication, and anterior gastropexy [1,2]. Endoscopic techniques have been presented; however, laparoscopic or open techniques are predominantly used [1]. While reduction can be achieved using endoscopic techniques, there is a high risk of recurrence thereafter [1]. To prevent recurrence, gastropexy and correction of any existing anatomic abnormalities are recommended [1]. In cases of incarcerated gastric volvulus, it is advisable to initiate treatment with an NGT or endoscopy for several days before proceeding with final surgery [1]. Repair of acute intrathoracic gastric volvulus can be safely and effectively accomplished through a laparoscopic approach [2]. To prevent volvulus and other complications after fundoplication, it is recommended that the stomach be fixed to both the hiatus and the peritoneum [1,4]. In infants and children, the established treatment for gastric volvulus involves immediate operative reduction, fixation of the stomach by gastropexy or gastrostomy, and fundoplication in cases of associated structural defects or reflux disease [5].

Fourteen cases of gastric volvulus following laparoscopic fundoplication have been documented, with only five cases involving an intra-thoracic stomach. Table 1 presents the documented cases of gastric volvulus following fundoplication, encompassing patients ranging from 3.5 months to 90 years. The onset of symptoms related to gastric volvulus varied from seven days to four years post-surgery. Most cases were treated using an open approach, with four cases being addressed laparoscopically and one was commenced laparoscopically but necessitated conversion to an open procedure due to failure to resolve stomach twisting. Causes of gastric volvulus included a laxity of the supporting ligaments, intra-thoracic stomach, postoperative adhesions, the presence of a gastrostomy tube, or pregnancy. In our case, the cause of the volvulus could be related to the adhesions and intra-thoracic stomach. The presence of the stomach in the chest has consistently warranted surgical intervention due to the perceived high risk of severe complications if left untreated, such as acute volvulus with perforation, gangrene, or hemorrhage [6].

**Table 1:** The reported cases of gastric volvulus after fundoplication M: Male; F: Female; GERD: Gastroesophageal reflux disease; EGD: Esophagogastroduodenoscopy

Article	Age	Gender	Presentation	Fundoplication time	Reason for fundoplication	Surgery	Cause of volvulus
Savolainen et al., 2015 [1]	60	F	Abdominal pain, nausea, and vomiting	4 months	Giant hiatal hernia	Laparoscopy to open gastropexy	Primary volvulus “laxity of the supporting ligaments”
Golash, 2005 [2]	23	M	Abdominal and chest pain, retching	1 year	Unknown	Laparoscopy: Herniated part of the stomach and liver was reduced into the abdomen, adhesiolysis, hiatus repair with a polypropylene mesh. Nissen refundoplication	Adhesions intrathoracic
Fung et al., 2003 [3]	4	M	Vomiting	7 days	GERD complicated by recurrent aspiration pneumonia	Laparotomy stomach approximated to the parietal peritoneum and the posterior rectal sheath gastrostomy tube	-
Fung et al., 2003 [3]	3.5m	M	Incidental	37 months	Recurrent apnea and severe GERD	Laparotomy hernia was reduced and the hiatus was repaired. The Nissen fundoplication was revised and a gastrostomy.	Partial volvulus of the gastric fundus around the intact old plication site
Alhajjat and Kowdley, 2012 [4]	90	F	Abdominal pain, vomiting, and obstipation	3 months	Recurrent gastric volvulus, paraesophageal hernia	Laparotomy gastrostomy tube	Adhesions
Kuenzler et al., 2003 [5]	13	M	Retching, emesis, and high gastrostomy output	4 months	Severe GERD	Laparotomy: The initial gastrostomy tube was removed and this site repaired. New gastrostomy tube.	Improper gastrostomy location
Baty et al., 2002 [7]	38	M	Abdominal pain, and vomiting	8 months	-	Laparotomy adhesiolysis	Adhesions
Yvergneaux et al., 1996 [8]	58	F	-	1 year	-	Laparotomy	Unknown intrathoracic
Chen et al., 1996 [9]	Child	-	-	Unknown	-	-	Improper gastrostomy location
Reyes-Zamorano, 2014 [10]	29	F	Abdominal pain, and vomiting	1 year	-	Laparoscopy release of adhesion band, Fastrorrhaphy, refundoplication, gastropexy	Adhesions
Reyes-Zamorano, 2014 [10]	40	F	Abdominal pain, and vomiting	6 months	-	Laparoscopy refundoplication	-
Brygger et al., 2013 [11]	38	F	Pregnant Abdominal pain, nausea and vomiting	6 months	GERD due to hiatus hernia	Caesarean section, followed by laparotomy diaphragmatic defect repaired.	Pregnancy intrathoracic
Idani et al., 2000 [12]	85	F	Nausea and vomiting	14 months		Laparotomy gastrotomy resection of the necrotic anterior wall of the stomach; Closure of the hiatus, and suturing of the stomach to the diaphragm.	Intrathoracic
Suwal et al., 2020 [13]	57	F	Abdominal pain and dysphagia	7 months	GERD, paraesophageal hiatal hernia	Laparoscopy: Subtotal linear gastrectomy with mediastinal abscess drainage and closure of the diaphragmatic defect was performed followed by intraoperative EGD	Intrathoracic

## Conclusions

Acute gastric volvulus after laparoscopic fundoplication is a surgical emergency and is challenging to diagnose. Early diagnosis is crucial to avoid fatal complications. A gastric volvulus should be used to differentiate and diagnose patients with recurrent vomiting after fundoplication. Initial efforts to decompress should involve nasogastric and gastroscopic procedures. The aim of surgery is to resolve the volvulus and prevent its recurrence. A laparoscopic approach is safe and feasible for repairing an acute intrathoracic gastric volvulus. The presence of the stomach in the chest requires surgical intervention due to the high risk of serious complications if left untreated.
